# Ocular expression of avian thymic hormone: changes during the recovery from induced myopia

**Published:** 2009-04-17

**Authors:** Jody A. Summers Rada, Allan F. Wiechmann

**Affiliations:** 1 Department of Cell Biology, University of Oklahoma Health Science Center, Oklahoma City, OK; 2 Oklahoma Center of Neuroscience, University of Oklahoma Health Science Center, Oklahoma City, OK; 3 Department of Ophthalmology, University of Oklahoma Health Science Center, Oklahoma City, OK

## Abstract

**Purpose:**

Several studies suggest that postnatal ocular growth is under the control of factors within the eye that regulate the rate of scleral extracellular matrix remodeling and the rate of ocular elongation. A microarray analysis was employed to identify some of the factors involved in the regulation of visually guided ocular growth. Gene expression was compared in the retina-retinal pigmented epithelium (RPE)-choroid of chick eyes that were decelerating in the rate of ocular growth (“recovering” from myopia) as compared with contralateral control eyes.

**Methods:**

Form-deprivation myopia was induced in the right eyes of two-day-old chicks by the application of translucent occluders. Following 10 days of deprivation, occluders were removed and chicks were provided unrestricted vision for an additional 1–7 days (recovery). After one and four days of recovery, chicks were sacrificed, retina, RPE, and choroid were isolated, and mRNA was subjected to microarray analysis using a chicken immune system 4000 gene microarray. In addition, whole eyes and isolated ocular tissues (retina and RPE, choroid, sclera, and extraocular muscle) of treated and control eyes were subjected to real-time PCR, immunohistochemistry, and western blot analyses to verify gene expression results.

**Results:**

Following one day of recovery, only one gene, avian thymic hormone (*ATH*) was highly upregulated (+12.3 fold). ATH gene and protein expression were confirmed in the retina and choroid as well as in the sclera and extraocular muscle. A significant increase in ATH protein was detected in choroids from treated eyes following four days of recovery as compared to contralateral controls (p<0.05; Wilcoxon signed-rank test).

**Conclusions:**

ATH is expressed in several ocular tissues and is specifically and rapidly (within one day) upregulated in the choroids of chick eyes recovering from induced myopia. This upregulation corresponds to the onset of choroidal thickening and increased choroidal vascular permeability. The identification of ATH in ocular tissues and its increased protein accumulation in the choroid during recovery from induced myopia suggest a novel role for this protein in the choroidal response to myopic defocus.

## Introduction

Myopia is a common ocular problem, affecting perhaps one billion people worldwide [1]. Most myopia is produced by lengthening of the vitreous chamber of the ocular globe, which generally stabilizes after puberty. However, failure of ocular elongation to stabilize results in scleral thinning, localized ectasia of the posterior sclera, retinal thinning, and possibly retinal detachment [2].

Animal models of myopia have provided significant insights into the cellular and molecular events underlying the control of ocular elongation and the refractive state. Depriving the retina of form vision by lid suture or translucent occluders (form deprivation), or by imposing hyperopic defocus on the retina with the use of minus lenses [3,4] results in an accelerated rate of axial elongation and a relative negative refractive error in the treated eye as compared with the contralateral untreated eye (relative myopia). In contrast, exposure to myopic defocus, as a result of restoration of unrestricted vision in previously form-deprived eyes (recovery) or treatment with plus lenses results in a decelerated rate of axial elongation and positive refractive error as compared with the contralateral untreated eye (relative hyperopia). Significant changes in scleral extracellular matrix synthesis, accumulation, and turnover are associated with changes in vitreous chamber elongation rates during the development of experimentally induced myopia or hyperopia in a variety of animals [5]. Although the mechanisms underlying visually guided changes in ocular elongation have yet to be determined, it is widely accepted that intrinsic factors within the eye play an important role in these scleral remodeling events [6]. Several laboratories have observed that the choroid undergoes dramatic changes in thickness and vascular permeability in response to changes in the visual environment [7–10]. When young chicks are fitted with translucent occluders to deprive the retina of form vision, choroidal thickness and permeability are reduced in the deprived eye, coincident with increased eye growth and myopia development. When the occluder is removed, and unrestricted vision is restored in chicks (“recovery”), choroidal thickness and vascular permeability are greatly increased and the rate of ocular growth in the treated eye is greatly decreased. We hypothesize that changes in choroidal thickness are the consequence of increased choroidal permeability [9]. These changes in choroidal thickness and permeability are suggested to provide a mechanism for compensating for induced changes in refractive error by moving the retina closer to the focal point of the eye [7]. Choroidal thickness and permeability changes may be the result of changes in choroidal blood flow [11], changes in the production of osmotically active molecules, such as glycosaminoglycans that draw water into lymphatic lacunae present in the choroidal stroma [7,12], or by the contraction and relaxation of nonvascular smooth muscle cells present within the choroidal stroma [13,14]. Moreover, these ocular growth changes are largely mediated by an unknown mechanism within the eye; changes in choroidal thickness and associated ocular growth changes occur in the absence of an intact optic nerve [15]. Similar changes in choroidal thickness have been observed in marmoset and macaques, but to a lesser degree [16,17].

While several recent reports have described gene and protein expression changes in retina and retina–retinal pigmented epithelium (RPE) associated with visually induced changes in ocular growth [18–20], no previous studies have examined gene or protein expression changes in the choroid during visually induced changes in ocular growth. Therefore, as a first step in elucidating any molecular mechanism associated with the choroidal response during the recovery from induced myopia, we employed microarray analyses to compare gene expression in chick retina together with retinal pigmented epithelium and choroid (retina–RPE–choroid) isolated from eyes experiencing myopic defocus as a result of prior form deprivation (recovering eyes) with contralateral untreated eyes. Using microarray analysis together with subsequent real time PCR, immunohistochemical and western blot analyses of the individual ocular tissues, for the first time we have identified the protein, avian thymic hormone (ATH), in several chick ocular tissues. Moreover, mRNA and protein levels of ATH appear to be significantly upregulated in the choroid during the recovery from induced myopia. Together, these results suggest that ATH may be involved in some aspect of the ocular changes associated with visually guided ocular growth.

## Methods

### Animals

White Leghorn cockerels (*Gallus gallus*) were obtained as two-day-old hatchlings from Ideal Breeding Poultry Farms (Cameron, TX). Form deprivation myopia was induced in two-day-old chicks by applying translucent plastic goggles as previously described [21,22]. Briefly, chicks were lightly anesthetized with isoflurane (Vedco Inc., St. Joseph, MO). Hemispheric goggles, cut from the bottoms of 15 ml, round bottom test tubes, were affixed to the feathers around the right eye of each chick with cyanoacrylate adhesive. Goggles remained in place for 10 days, after which time chicks were either sacrificed for isolation of ocular tissues from control and form-deprived eyes, or goggles were removed and chicks were allowed to experience unrestricted vision (recover) for one, four, or seven days. The left eyes of all the chicks were never covered and were used as controls. Previous studies have demonstrated that the rate of glycosaminoglycan synthesis in the posterior sclera and the rate of ocular elongation are significantly increased following two days form deprivation [22,23]. Upon restoration of unrestricted vision (10−14 days of form deprivation), glycosaminoglycan synthesis in the posterior sclera and the rate of ocular elongation drop to their lowest levels following four and seven days of recovery, respectively [9,22]. Based on these previous studies, the time courses for the present study were designed to identify molecular changes in ocular tissues induced early in the recovery process, as well as changes that are manifested when the eye has dramatically slowed its rate of vitreous chamber elongation and has demonstrated choroidal thickening. Additionally, ocular tissues were harvested from untreated normal eyes of 10-day-old chicks for comparison of ATH expression in ocular tissues (n=3).

Birds were housed in temperature-controlled brooders on a 12 h:12 h light-dark cycle, allowed food and water ad libitum, and checked twice a day. The chicks were maintained and used in accordance with the Animal Welfare Act and National Institutes of Health Guidelines and the ARVO Statement for the Use of Animals in Ophthalmic and Vision Research. All procedures adhered to the Institutional Animal Care and Use Committee of the University of Oklahoma Health Sciences Center.

### Microarray analyses

For microarray analyses, retina–RPE–choroid complexes were isolated and pooled separately from six treated and contralateral control chick eyes following one and four days of recovery. This was done by using TRIzol reagent according to the manufacturer’s instructions (Invitrogen, Carlsbad, CA) and RNeasy MinElute CleanUp (Qiagen, Valencia, CA). RNA was then amplified and labeled using Amino Allyl message Amp aRNA kit (Qiagen). RNA concentration and purity were determined by ultraviolet spectrophotometry at 260 nm and 280 nm and then transferred for microarray analysis to the Fred Hutchinson Cancer Research Center (FHCRC) Genomics Resource DNA Array Laboratory (Seattle, WA). Following amplification and labeling of RNA, microarray analyses were conducted using a chicken immune system 4000 gene microarray (FHCRC Genomics Resource).

Production and use of this chicken immune system glass slide cDNA microarray have been previously described [24,25]. Briefly, the array was composed of clones from three expressed sequence tag (EST) libraries: about 1,800 clones from the DT40subNB library derived from the DT40 bursal lymphoma cell line, about 1,200 clones from a chicken-activated T-cell library [26], and about 500 clones from a normal two-week bursal library, DKFZ426 [27]. The complete array contained 3451 cDNAs representing about 2,700 different genes. Since cDNAs were derived from EST libraries, clones with the same gene identification usually contained different partial cDNA sequences and were located in different portions of the spotted array. The different representatives of the same gene served to help determine experimental consistency of expression results for individual genes of interest and were therefore included in the summary of microarray results (Table 1).

**Table 1:** Real-time PCR gene primers.

**Gene**	**GenBank**	**Primer**
Avian Thymic Hormone (*ATH*)	M94894.1	F: TGAAGGCATAACAGCGTGAG
		R: GGATGGAAAGCCTGAACAAA
Glyceraldehyde 3-phosphate dehydrogenase (*GAPDH*)	NM_204305	F: TGCTAAGGCTGTGGGGAAAGTC
		R: CAAAGGTGGAGGAATGGCTGTC

Reference Cy5 dye-labeled cDNA from retina/RPE/choroid complexes for 1-day recovering eyes was mixed with Cy3 labeled cDNA from contralateral control eyes and simultaneously hybridized to microarrays under glass coverslips for 16 h at 63 °C. Additionally, dye swap control experiments were performed in which Cy3 dye-labeled cDNA from retina/RPE/choroid complexes for 1-day recovering eyes was mixed with Cy5 labeled cDNA from contralateral control eyes before hybridization. Fluorescent array images were collected for both Cy3 and Cy5 by using a GenePix 4000A fluorescent scanner (Axon Instruments, Foster City, CA), and image intensity data were extracted and analyzed by using Genepix pro 3.0 analysis software. Normalization of the Cy5 to Cy3 signal in each experiment was determined by assuming equal global hybridization of test and reference probes to DKFZ426 and T-cell library clones on the array. Genes in the retina–RPE–choroid of treated eyes that exhibited ≥ 2.5 fold expression differences (upregulated or downregulated) were considered significant and included in Appendix 1.

### Real-time PCR

Total RNA was extracted from isolated retina–RPE, choroid, sclera, and extraocular muscle of both eyes of three normal 10-day-old chicks. Extraocular muscle was included as a positive control for ATH expression, since parvalbumins are known to be expressed in skeletal muscle and ATH is considered a parvalbumin [28–30]. Briefly, 5 mm punch biopsies were obtained from the posterior pole of each eye in such a way that they contained retina, RPE, choroid, sclera, and extraocular muscle. These were placed in a Petri dish containing phosphate buffered saline (137 mM NaCl, 2.7 KCl, 4.3 mM Na_2_HPO_4_, 1.47 mM KH_2_PO_4_, pH 7.4; PBS). With the aid of a binocular dissection scope, the retina and RPE were removed as a sheet from the 5 mm punch with a small spatula and snap froze the sheets in microfuge tubes in liquid nitrogen. Occasionally, small pieces of RPE would adhere to the choroid, and these were removed by gentle brushing with a sable brush and rinsing with PBS. PBS containing the RPE was collected and added to tubes containing retina–RPE. Forceps were used to remove the choroid and extraocular muscle from the scleral punches, and choroid, sclera, and muscle were placed separately in microfuge tubes and snap frozen in liquid nitrogen. The dissection of the sclera from extraocular muscle is straightforward since the sclera consists of cartilage, which is readily and effectively isolated from the extraocular muscle and other ocular tissues. However, we cannot rule out the possibility of cross contamination of mRNA between some ocular tissues, such as the RPE and choroid. Total RNA was isolated from each ocular tissue using TRIzol reagent (Invitrogen) and RNeasy MiniElute CleanUp (Qiagen). Scleral punch biopsy specimens were pulverized under liquid nitrogen using a Dounce tissue grinder mounted to a drill. All tissues (retina–RPE, choroid, pulverized sclera and extraocular muscle) were then homogenized in 1 ml TRIzol Reagent using a VirTis homogenizer (Gardiner, NY) before 5 min incubation at room temperature (RT) and 200 μl chloroform treatment for dissociation of nucleoprotein complexes. The tubes were vigorously shaken by hand for 15 min, then incubated at RT for 2 min, before they were centrifuged at 12,000x g for 15 min at 4 °C. Following centrifugation, the upper aqueous (RNA) phase was collected into a fresh tube then precipitated into a gel-like pellet with 500 μl isopropyl alcohol by incubating for 10 min at RT and centrifugation at 12,000x g for 15 min at 4 °C. After removal of the supernatant, the RNA pellet was washed once with 1 ml 75% ETOH by centrifugation at 7,500x g for 5 min at 4 °C. The RNA pellet was dried by air for 10 min before the RNA was dissolved in 100 μl RNase-free water and incubated for 10 min at 60 °C. RNA was stored at −80 °C until use. RNA concentration and purity were determined at an optical density ratio of 260:280 using the Nanodrop® ND-1000 spectrophotometer (NanoDrop Technologies, Wilmington, DE). cDNA was generated from total RNA by reverse transcription, and real-time PCR was performed as previously described [28]. Briefly, samples from each bird were analyzed in triplicate using gene-specific chicken primers together with SYBR Green (Molecular Probes, Eugene, OR) in a 96 well plate format, using an i-Cycler iQTM Multi-Color Real Time PCR Detection System (Bio-Rad, Hercules, CA). Primers were selected from chick sequences of *ATH* and glyceraldehyde 3-phosphate dehydrogenase (*GAPDH*) using BLAST and Primer3 and ordered from Sigma-Genosys (St. Louis, MO; Table 1). The chicken *GAPDH* gene was used as a control to normalize for variation in starting cDNA between samples. For both *ATH* and *GAPDH*, denaturation was performed for 45 s at 95.0 °C, primer annealing for 45 s at 62 °C, and extension was performed for 60 s at 72.0 °C. Relative levels of *ATH* gene expression were determined for the retina–RPE, choroid, sclera, and extraocular muscle using the mean normalized expression (MNE) values as previously described [28,29]. Briefly, MNE values are calculated as the ratio of the efficiency and mean threshold cycles of the PCR reaction of the reference gene, G3PDH, to the efficiency and mean threshold cycles of the target gene, ATH. The MNE is calculated from an exponential equation where the values for the efficiencies of the reference and target genes serve as the base and the mean cycle thresholds of the reference and target gene are the exponents. In this method, the expression levels of the gene of interest can be normalized to a housekeeping gene to correct for differences in starting mRNA concentrations between samples. Correct product size was confirmed by DNA agarose gel, and lack of primer dimer formation was verified by melt curve analysis.

### Immunohistochemistry

Immunohistochemical detection of ATH was performed as follows. One chick was form vision-deprived in the right eye for 13 days followed by a four-day period of unrestricted vision in the treated eye. At the end of the recovery period, the chick was anesthetized with 0.8% isoflurane (Vedco Inc.) inhalation anesthesia in oxygen then perfused through the left ventricle with approximately 1,000 ml PBS, pH 7.4, at roughly18–20 °C to clear blood from ocular tissues. After the perfusion, the eyes were enucleated, opened at the equator, and a 5 mm punch biopsy that contained retina, RPE, choroid, sclera, and extraocular muscle was obtained at the posterior pole of the treated and contralateral control eyes. Ocular tissue punches were fixed in 4 °C in 4% paraformaldehyde in 0.1 M phosphate buffer pH 7.4, followed by immersion in 30% sucrose in phosphate buffer for 16–20 h at 4 °C. Tissue punches were then embedded and frozen in O.C.T. embedding compound (Tissue-Tek, Elkhart, IN). Serial cross-sections of each tissue punch were cut into 10-μ-thick sections using a cryostat microtome and collected on glass slides.

For immunocytochemical localization of ATH in chick ocular tissues, cryostat sections were rinsed in PBS, and then incubated for 30 min at RT in incubation buffer that consisted of 1% BSA (Sigma), 0.2% Triton X-100, and 0.004% sodium azide in PBS. Sections were incubated overnight at 4 °C with mouse anti-ATH monoclonal antibody (obtained as a generous gift from Dr. Michael Henzl, University of Missouri-Columbia, Department of Biochemistry, Columbia, MO) diluted 1:500 in incubation buffer. For negative controls, tissue sections were incubated in 2 μg/ml nonimmune mouse immunoglobulin (Sigma) instead of the ATH antibody. Additional preabsorption controls were performed in which the anti-ATH antibody was incubated overnight at 4 °C with 2 μM of purified chicken ATH [30] (also obtained as a generous gift from Dr. Michael Henzl) before immunolabeling fixed cryostat sections of chick ocular tissues. Following overnight incubation with the primary antibody, sections were rinsed in PBS, and incubated for 30 min at RT in 5 μg/ml of AlexaFluor 488 (green) or AlexaFluor 568 (red) conjugated to rabbit anti-mouse antibody (Molecular Probes). Sections were rinsed in PBS and then incubated for 10 s at RT with 0.0005% DAPI nuclear stain, followed by a final rinse in PBS. Coverslips were mounted onto the slides with Prolong Gold Antifade reagent containing DAPI (Invitrogen), and the immunolabeled sections were examined under an Olympus Fluoview 1000 laser-scanning confocal microscope (Center Valley, PA). The anti-ATH antibody used in these studies has been previously demonstrated to be specific for ATH, and does not cross-react with other chicken parvalbumins [30].

### Western blot analysis

Chick retina–RPE, choroid, and sclera were isolated separately from 5 mm punch biopsy specimens obtained from the posterior poles of form-deprived eyes, recovering eyes (one, four, and seven days), contralateral control eyes, and normal eyes (n=3 treated and contralateral control eyes for each condition). Total protein was extracted separately from each tissue by vigorous mixing in 100 μl/extract of 2% SDS. Suprachoroidal fluid was collected from the central posterior pole of enucleated chick eyes as previously described [10]. This was done by inserting the needle, bevel side up, just beneath the sclera, in the suprachoroidal space and withdrawing 10–40 µl of fluid from each eye. A larger volume (30–40 μl) of suprachoroidal fluid was generally obtained from recovering eyes, while 10–20 μl of fluid was usually obtained from control and form-deprived eyes. Individual suprachoroidal fluid samples were centrifuged at 16,870x g at 4 °C for 15 min to pellet blood cells and any cellular debris. However, it is possible that cells damaged during collection of suprachoroidal fluid could have released cellular contaminants into the fluid that may not be removed by centrifugation. Total protein in individual tissue extracts and suprachoroidal fluid was determined by employing the microBCA protein assay with a 96 well plate format (Pierce Biotechnology, Rockford, IL). Protein concentration was determined by measuring absorbance at 570 nm with comparison to protein standards (BSA; 0.5–200 μg/ml). Aliquots of protein extracts (8 μg total protein) were directly applied to 10% Bis-Tris Gel NuPAGE™ SDS–PAGE gels (Invitrogen). Gel samples were electrophoresed under reducing conditions and electroblotted onto a nitrocellulose membrane using an electro-transfer unit (XCELL Sureback™ Electrophoresis Cell, Invitrogen) or stained with SimplyBlue™ SafeStain (Invitrogen) according to the manufacturer’s instructions. Blots were probed for 4 h at RT with mouse anti-chicken ATH monoclonal antibody at a 1:500 dilution in blocking buffer composed of PBS containing 0.1% Tween-20 and 0.2% I-Block (Tropix, Bedford, MA). This was followed by incubation with rabbit anti-mouse IgG (whole-molecule) conjugated to alkaline phosphatase (Sigma) at a dilution of 1:1,000 for 1 h at RT. Between incubations the blot was washed three times for 10 min per wash with 1× PBS containing 0.05% Tween-20. CDP-*Star* ® Ready-to-Use with Nitro-Block II™ (Tropix) was added to the blot for 5 min and then exposed on film. For quantification of ATH, film exposures were adjusted for each blot (each tissue) to be in the linear range of detection. Digitized images of the developed films were obtained using a flatbed scanner, and densitometry was performed using NIH Image version 1.63 (National Institutes of Health, Bethesda, MD).

### Statistical analysis

Comparisons of ATH protein expression differences between treated and contralateral control eyes in five treatment groups were made using the Kruskal–Wallis ANOVA nonparametric test for mean treatment effect across groups. Additionally, pairwise testing using the Wilcoxon signed-rank tests was used to measure ATH expression between pairs of treated and control eyes. Statistical analyses were performed with the assistance of GraphPad Prism version 4.03 for Windows (GraphPad Software, San Diego, CA) and GB-STAT for Macintosh (Dynamic Microsystems, Inc., Silver Spring, MD).

## Results

### Microarray analysis of retina–RPE–choroid during recovery

Analysis of the 4,000 gene microarrays identified a total of 30 loci representing 14 genes which were either upregulated or downregulated in experimental eyes by at least a 2.5 fold change over contralateral control eyes (Appendix 1). Following one day of recovery, only the expression of one gene on our microarray, *ATH* (accession number M94894.1), was highly upregulated (+12.3 fold) and no genes were downregulated in their expression by ≥2.5 fold. Following four days of recovery, 10 genes were downregulated and one gene, ovotransferrin, was upregulated.

### *ATH* expression in ocular tissues

**Figure 1:**
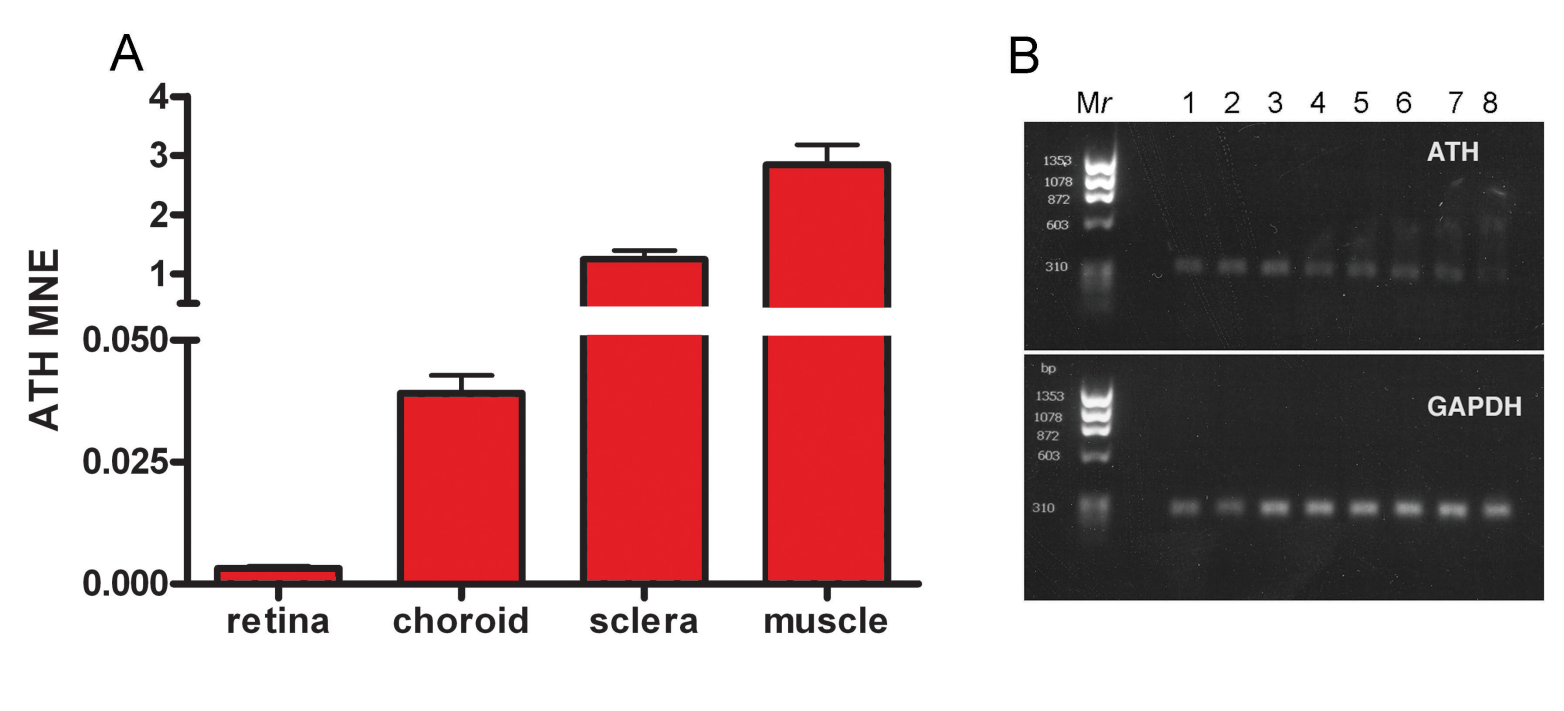
Real time RT–PCR quantification of *ATH* in chick ocular tissues. Steady-state levels of *ATH* mRNA were measured in the retina–RPE, choroid, sclera, and extraocular muscle using chick gene-specific primers (Table 1). **A:** The average mean normalized expression (MNE) of ATH was calculated in each ocular tissue of three untreated 10 day old chicks. All reactions were run in triplicate and normalized to the reference gene, *GAPDH*. **B:** Shown are ethidium bromide gels of real-time PCR products of *ATH* and *GAPDH* in chick extraocular muscle over temperatures ranging 66–55 °C. M*r*: PhiX174RF DNA/HaeIII molecular weight ladder, Lane 1: 66.0 °C, Lane 2: 65.2 °C, Lane 3: 63.9 °C, Lane 4: 61.8 °C, Lane 5: 59.0 °C, Lane 6: 57.2 °C, Lane 7: 55.8 °C, Lane 8: 55.0 °C.

Due to the relatively high level of upregulation of *ATH* in the retina–RPE–choroid of chick eyes following one day of unrestricted vision (Appendix 1), and since *ATH* expression had not been previously identified in ocular tissues, real-time PCR was used to compare relative levels of *ATH* mRNA in chick retina–RPE, choroid, sclera, and extraocular muscle (Figure 1A). Expression levels of *ATH* were calculated in chick retina–RPE, choroid, sclera and extraocular muscle with the cycle threshold values normalized to the housekeeping gene, *GAPDH* using the MNE. *ATH* expression levels were highest in extraocular muscle (MNE=2.85±0.335) and sclera (1.24±0.149), and substantially lower in the retina–RPE (0.0032±0.0004) and choroid (0.039±0.0036). Ethidium bromide-stained agarose gels of real PCR products were run to verify the correct size and amplification of *ATH* and *GAPDH* in chick extraocular muscle over temperatures ranging 66–55 °C (Figure 1B). Although isolation of the retina–RPE, choroid, sclera, and extraocular muscle is straightforward, the possibility exists for cross-contamination of cellular material and mRNA between the RPE and choroid, thereby altering the relative levels of *ATH* mRNA in these tissues somewhat from that which occurs in vivo.

### ATH protein in chick ocular tissues

**Figure 2:**
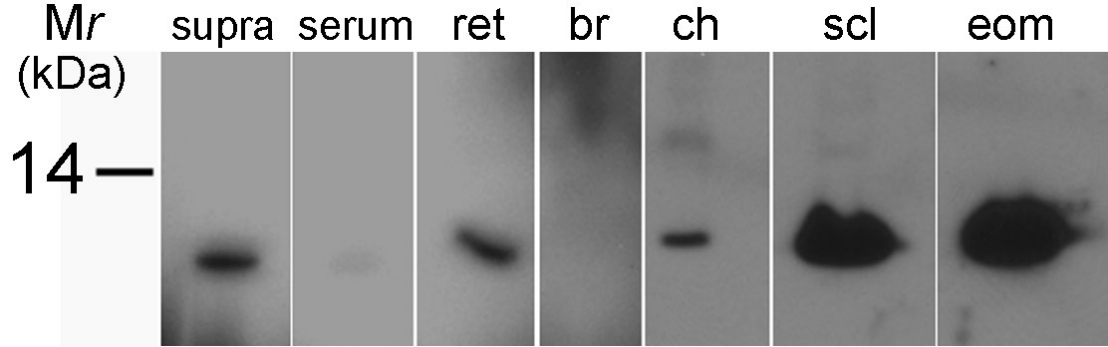
ATH protein detection in the chick eye. Western blot analysis identified the presence of ATH in the suprachoroidal fluid (supra), retina–RPE (ret), choroid (ch), sclera (scl), and extraocular muscle (eom) of the chick eye. An ATH-immunopositive band was barely detectible in serum (serum), and no ATH was detected in chick brain (br). Total protein from each sample was determined using the BCA assay, and 8 μg total protein was loaded into each well.

A specific monoclonal antibody generated against chicken ATH (Michael Henzl, University of Missouri-Columbia, Department of Biochemistry, Columbia, MO) was used in western blot analysis to examine the distribution of ATH in the suprachoroidal fluid, serum, retina, brain, choroid, sclera, and extraocular muscle of the chick eye (Figure 2). A single band of approximately 11.5 kDa could be identified in chick protein extracts of choroid, retina, sclera, and extraocular muscle. No labeling was observed of bands at *M*_r_ other than 11.5 kDa (Figure 2). Additionally, ATH was detected in suprachoroidal fluid. ATH was nearly undetectable in serum, and no ATH immunopositive bands were seen in the chick brain. Although very low levels of ATH were detected in the serum in the present study, a previous study identified ATH in the blood of chicks [31]. Therefore, for immunolocalization studies, chicks were perfused with approximately 1,000 ml PBS to clear blood from ocular tissues.

**Figure 3:**
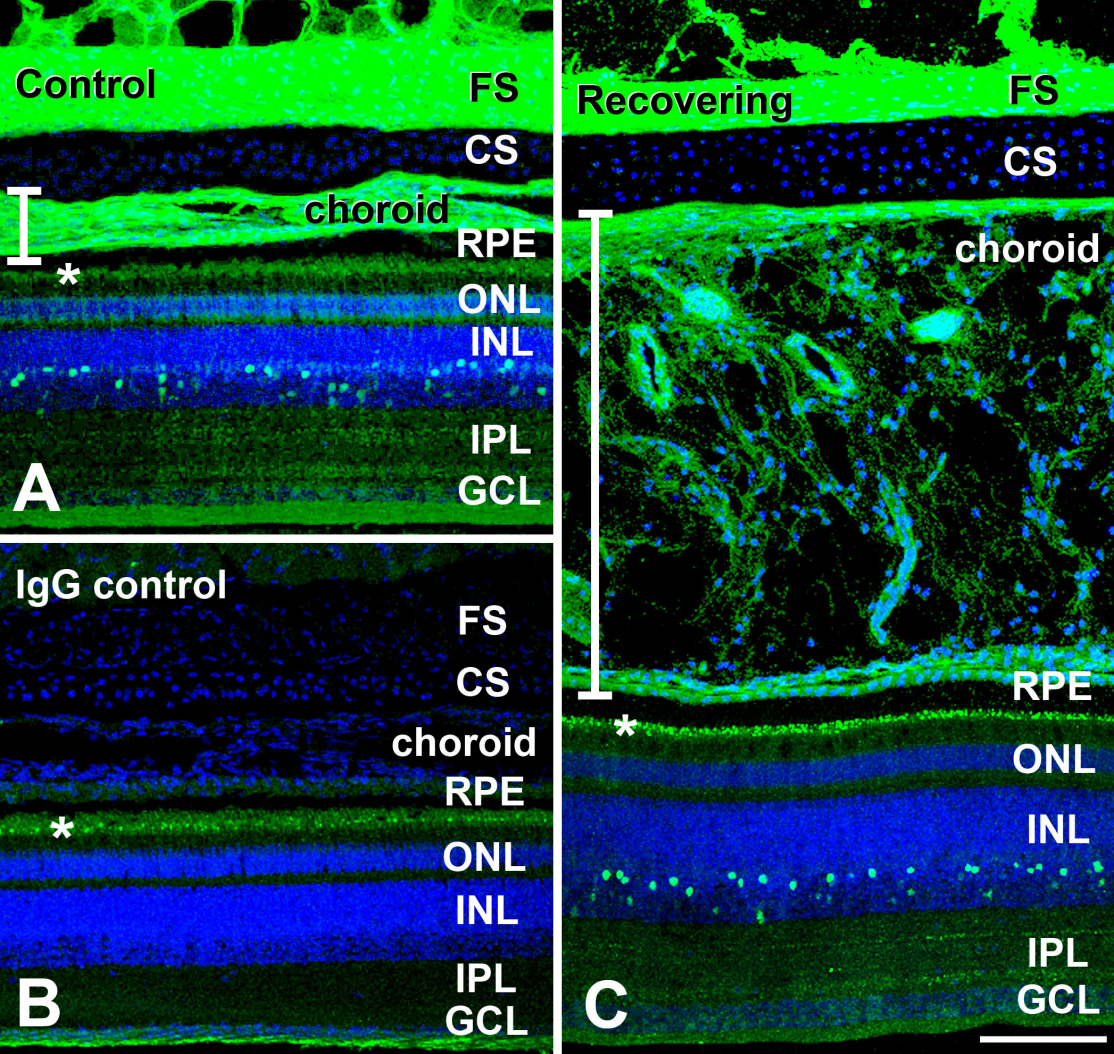
Localization of Avian Thymic Hormone (ATH) labeling in posterior ocular tissues of control and recovering chick eyes. Monoclonal antibodies specific for ATH (obtained from Michael Henzel, University of Missouri, Columbia, MO) were used together with AlexaFluor 488-conjugated rabbit anti-mouse immunoglobulin to localize ATH in chick ocular tissues. **A:** ATH immunolabeling of a control eye demonstrated intense labeling in the fibrous scleral layer (FS) and the choroid. Additionally, ATH was detected in a subpopulation of cell bodies located in the inner nuclear layer (INL) and in two sublaminae of the inner plexiform layer, most likely representing the synaptic processes of ATH-positive cells of the INL. **B:** IgG control section of a control eye is shown where non-immune mouse IgG was used in the first incubation, followed by incubation in AlexaFluor 488-conjugated rabbit anti-mouse immunoglobulin. Fluorescence of lipid droplets in the photoreceptor inner segments (*) and in the nerve fiber layer is nonspecific. Nuclei were stained with DAPI (blue). **C:** ATH distribution in a recovering eye was similar to that of controls, with specific immunolabeling in the fibrous sclera, as well as throughout the markedly thickened choroid, cells of the INL and sublaminae of the inner plexiform layer (IPL). Vertical bars indicate thickness of choroid layer in control (3A) and recovering (3C) eyes. Abbreviations: cartilaginous sclera (CS), outer nuclear layer (ONL), ganglion cell layer (GCL). Scale bar (**A–C**) equals 100 μm.

**Figure 4:**
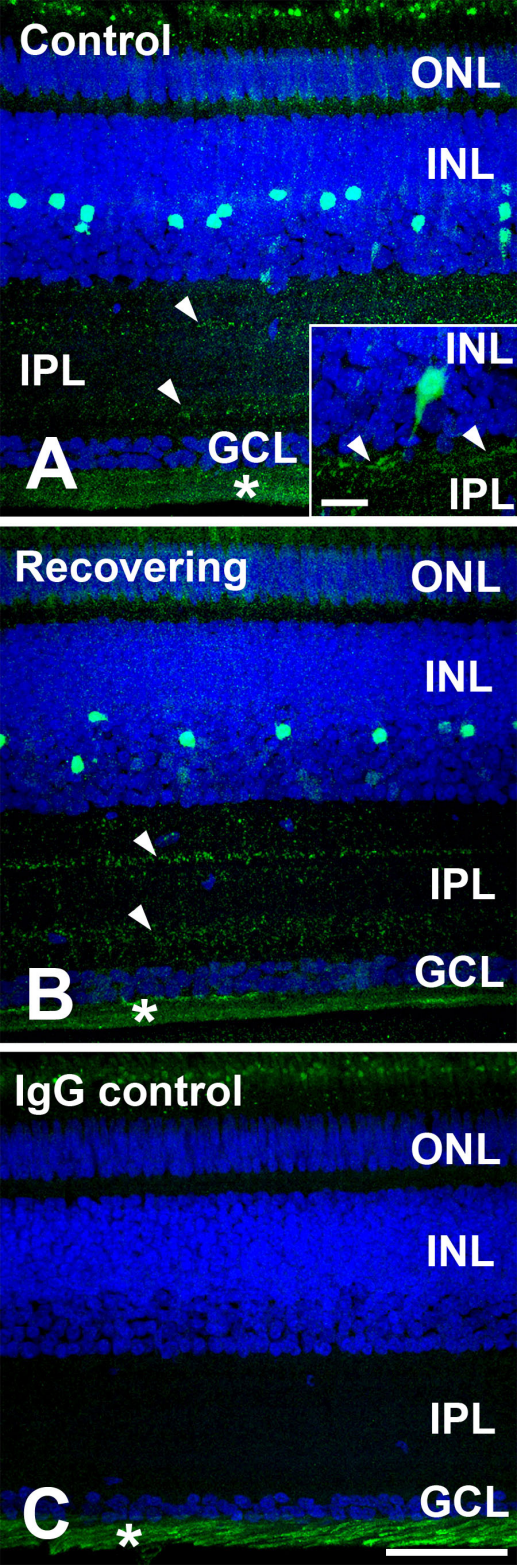
Distribution of ATH in the retina of control and recovering chick eyes. **A:** ATH immunolabeling in the retina of a control chick eye ATH immunoreactivity can be seen in a subpopulation of amacrine cells in the inner nuclear layer (INL) as well as in proximal and distal sublaminae in the inner plexiform layer (IPL; arrowheads). Nonspecific labeling is present in lipid droplets in the photoreceptor inner segments and in the nerve fiber layer (*). Inset: A presumptive ATH-immunoreactive bistratified amacrine cell is demonstrated with a process appearing to extend to the distal IPL (arrowhead). Scale bar represents 50 μm. **B:** ATH immunolabeling is shown in the retina of a recovering chick eye. Similar to the control eye, ATH immunoreactivity can be seen in a subpopulation of amacrine cells in the INL as well as in proximal and distal sublaminae in the IPL (arrowheads). **C:** IgG control section of the retina of a control eye is shown, where non-immune mouse IgG was used in the first incubation, followed by incubation in AlexaFluor 488-conjugated rabbit anti-mouse immunoglobulin. Scale bar (**A–C**) represents 50 μm.

**Figure 5:**
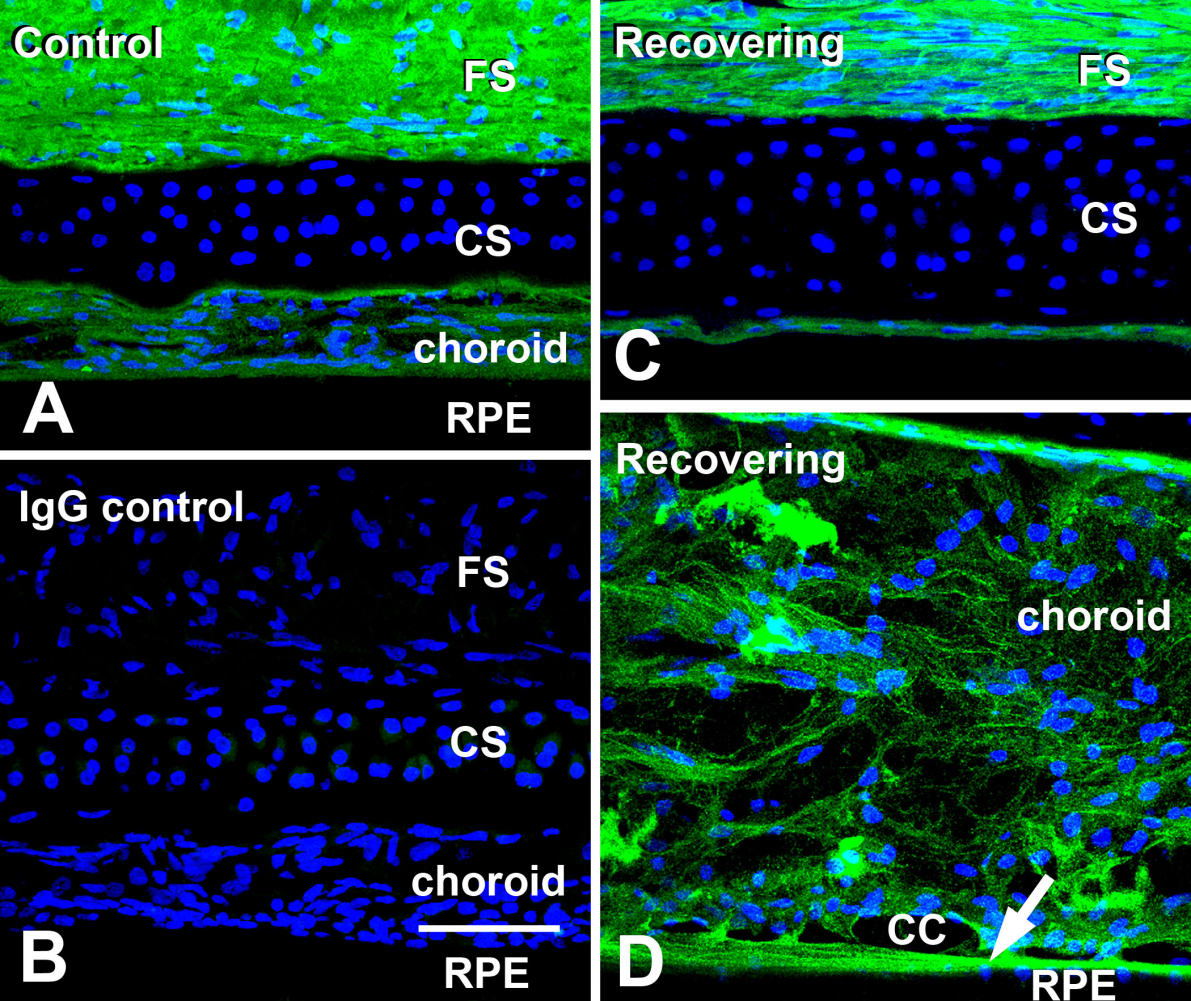
Distribution of ATH in the sclera and choroid of control and recovering chick eyes. Monoclonal antibodies specific for ATH were used together with AlexaFluor 488-conjugated rabbit anti-mouse immunoglobulin to localize ATH in sclera and choroid of control and recovering eyes. **A:** Intense ATH immunolabeling is detected in the fibrous sclera (FS) and in perivascular and extravascular regions of the choroid. ATH was absent in the cartilaginous scleral layer (CS). **B:** IgG control section of the sclera and choroid of a control eye, where nonimmune mouse IgG was used in the first incubation, followed by incubation in AlexaFluor 488-conjugated rabbit anti-mouse immunoglobulin. **C:** ATH immunolabelling in the sclera of a recovering chick eye is shown in a region artifactually separated from the choroid. ATH can be seen localized in the outer fibrous sclera (FS) as well as in the thin inner fibrous sclera on the choroidal side of the sclera. **D:** ATH immunolabeling in the choroid of a recovering chick eye is shown. ATH can be seen throughout the stroma of the markedly expanded choroid and on the choroidal side of Bruch’s membrane (arrow), but is absent in the RPE. Nuclei were stained with DAPI (blue). Choriocapillaris is abbreviated CC. Scale bar (**A–D**) represents 50 μm.

**Figure 6:**
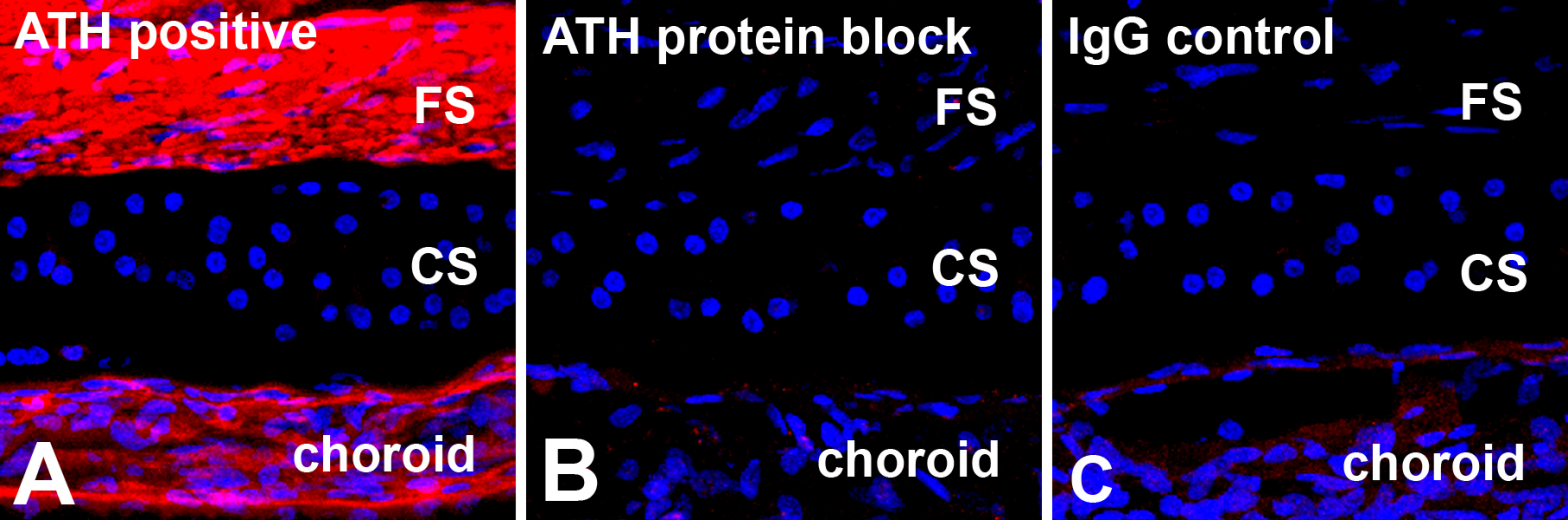
Specificity of anti-ATH on sections of chick ocular tissues. Fixed cryostat sections of chick sclera and choroid were incubated with **A**) monoclonal anti-ATH antibody (ATH positive), with **B**) anti-ATH antibody that was preincubated with 2 μM recombinant chick ATH (ATH protein block), or with **C**) nonimmune mouse IgG (IgG control) followed by incubation in anti-mouse IgG conjugated to AlexaFluor 568 (red). No specific immunoreactivity is observed in fibrous sclera (FS) and choroid when antibodies were preincubated with ATH, indicating the specificity of the anti-ATH antibodies for ATH in chick ocular tissues, cartilaginous sclera is abbreviated as CS.

Confocal microscopy enabled detection of specific immunolabeling for ATH in the retina, choroid, and fibrous layer of the chick sclera (Figure 3 and Figure 4). ATH immunolabeling was mostly detected in cell bodies located on the inner 1/3 to 1/2 of the inner nuclear layer (INL) of the retina. Punctate labeling was also detected in the proximal and distal sublaminae of the inner plexiform layer (IPL) which most likely represents the synaptic processes of ATH-positive cells of the INL. In some cases, ATH labeling could be seen in cell processes extending from the amacrine cell bodies to the synaptic terminals in the IPL (Figure 4A, inset). Comparison with nonimmune IgG controls indicated that ATH-positive labeling in the photoreceptor inner segments represented nonspecific labeling of lipid droplets possibly due to absorption of the fluorophore into the lipid matrix of the droplets or nonspecific immunolabeling (Figure 3). ATH labeling was observed throughout the choroidal stroma, the choriocapillaris, and choroidal portion of Bruch’s membrane of control and recovering eyes (Figure 3B and Figure 5C). In recovering eyes, the choroid was substantially thickened, and ATH could be seen distributed throughout the choroidal stroma (Figure 3C and Figure 4D). Comparison with negative controls indicated that ATH was not present in the RPE or the RPE portion of Bruch’s membrane. However, the presence of intracellular ATH in the RPE cannot be excluded due to the presence of pigment in mid- to apical parts of the cells which might obscure ATH immunoreactivity. Intense ATH labeling was detected in the fibrous layer of the chick sclera, but not in the cartilaginous layer (Figure 3 and Figure 5). To verify the specificity of the anti-ATH antibodies for ATH in chick ocular tissues, we preincubated ATH antibodies with 2 μM chick ATH before immunolabeling (Figure 6B, ATH protein block) and compared immunolabeling patterns were compared between sections treated with anti-ATH (Figure 6A), ATH protein block (Figure 6B), and nonimmune mouse IgG (Figure 6C). No immunoreactivity was observed in fibrous sclera, choroids, and retina (not shown) using ATH protein blocked antibodies, indicating the absence of nonspecific immunolabeling by anti-ATH in chick ocular tissues.

### Changes in ATH protein during recovery from induced myopia

**Figure 7:**
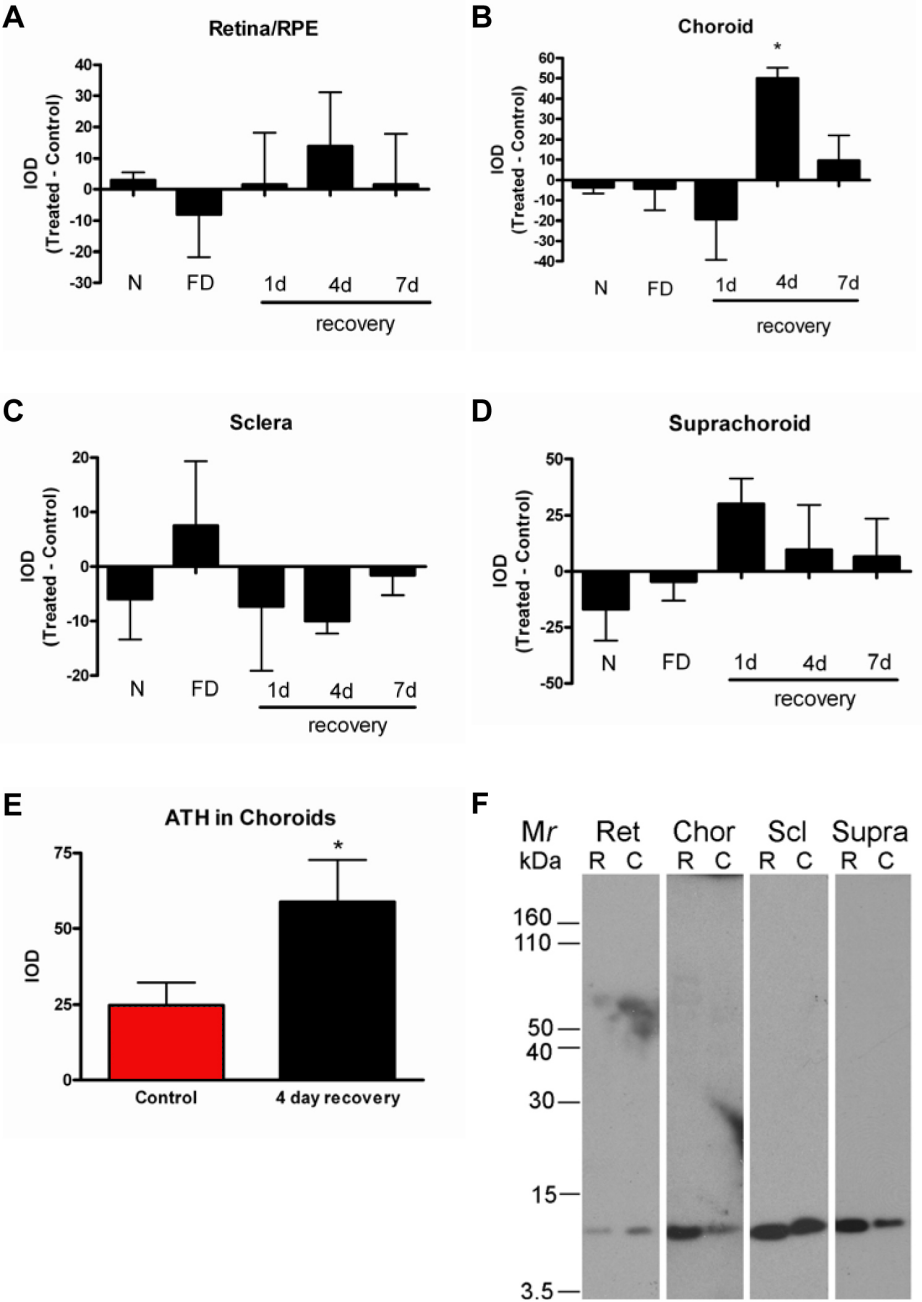
ATH protein accumulation in posterior chick ocular tissues from eyes in various growth states. Retina–RPE, choroid, sclera, and suprachoroidal fluid were harvested from the posterior poles of 10-day-old untreated, normal eyes (N), following 10 days of form deprivation (FD), and following 1, 4, and 7 days of unrestricted vision with prior form deprivation for 10 days (1 day, 4 day, and 7 day recovery, respectively) together with tissues from contralateral control eyes. Total protein (8 μg) from each tissue extract was separated by SDS–PAGE, and ATH protein expression was detected by western blot analysis. The 11.5 kDa band from each sample was quantified by densitometry using NIH Image v. 1.63. Bars represent the integrated optical density (IOD) of ATH bands on western blots from treated eyes minus IOD of contralateral controls (mean±SEM). **A:** ATH protein expression was measured in the posterior retina/RPE. **B:** ATH protein expression was measured in the posterior choroid. A large increase in ATH was detected in choroids following 4 days of recovery as compared with ATH levels in choroids of contralateral controls. **C:** ATH protein expression was measured in the posterior sclera. **D:** ATH protein accumulation was measured in aliquots of suprachoroidal fluid isolated from the posterior poles of enucleated eyes. **E:** ATH protein expression was measured in choroids following 4 days of recovery and in contralateral control eyes (n=6 additional pairs of control and treated eyes). **F:** Representative western blots are shown of ATH protein in retina/RPE (Ret), choroid (Chor), sclera (Scl), and suprachoroidal fluid (Supra) in treated (R) and control (C) eyes following 4 days of recovery. Data represent the mean±SEM for n=3 birds (3 pairs of control and treated eyes for figures **A-D** and n=6 pairs for figure **E**) in each group *p<0.05. Comparisons between recovering and contralateral control eyes were made using the Wilcoxon signed-rank test for paired data.

Total accumulated ATH was measured in retina–RPE, choroid, suprachoroidal fluid, and sclera of normal, untreated chick eyes, treated eyes following 10 days of form deprivation, and following one, four, or seven days of unrestricted vision with prior form deprivation for 10 days (recovery), and contralateral control eyes (Figure 7A-F). Equivalent amounts of total protein (8 μg/lane) extracted from tissues or suprachoroidal fluid were electrophoresed and transblotted onto nitrocellulose for Western detection using anti-ATH together with anti-mouse IgG conjugated to alkaline phosphatase. Using this method, we could detect purified chicken ATH [30] in the linear dynamic range from 20 to 500 ng (data not shown). Representative blots of ATH in retina–RPE, choroid, sclera, and suprachoroidal fluid in control and treated eyes following four days of recovery are shown in Figure 7E. A substantial increase in ATH was detected in choroid extracts from treated eyes following four days of recovery as compared to contralateral controls (p<0.05; Wilcoxon signed-rank test). The increase in ATH expression in treated eyes following 4 days of recovery was significantly different from that of all other treatment groups (p<0.05; Kruskal–Wallis ANOVA) however, due to the small sample size used (n=3), important treatment differences in other groups may have gone undetected (Figure 7B). No significant differences were detected in ATH concentration in treated eyes as compared with controls in retina–RPE (Figure 7A), sclera (Figure 7C), or suprachoroidal fluid (Figure 7D) of normal eyes, form deprived eyes, or recovering eyes. Choroidal expression of ATH was re-evaluated by western blot analyses in six additional birds following four days of recovery as compared with contralateral controls to verify increased expression in treated eyes as compared with controls (p<0.05; Wilcoxon signed-rank test) (Figure 7E).

## Discussion

In an attempt to elucidate the retina-to-sclera chemical cascade involved in emmetropization, we examined changes in retina–choroid–RPE gene expression in eyes in which the rate of ocular growth was dramatically slowed due to imposed myopic defocus as a result of prior form deprivation (recovery from form deprivation myopia). Analysis of a 4,000 gene microarray identified the expression of one gene, *ATH*, to be upregulated in the retina–RPE–choroid following one day of recovery as compared with contralateral control eyes. The elevation of *ATH* gene expression following one day of recovery suggests that this hormone is involved in immediate retinal–choroidal signaling events initiated by restoration of unrestricted vision. Following four days of recovery, one gene, ovotransferrin, was significantly upregulated in the retina–RPE–choroid of recovering eyes as compared with contralateral control eyes. This finding is consistent with our previous finding that ovotransferrin protein synthesis is significantly increased in isolated choroids during the recovery from induced myopia [9]. The consistency of these two independent findings supports the validity of these microarray results. No genes were downregulated following one day of recovery. However, following four days of recovery, 10 genes were downregulated by ≥ 2.5 fold. The finding that several housekeeping genes including *GAPDH*, heat shock cognate 70, and ribosomal protein large P0 were downregulated by ≥ 2.5 fold at multiple loci on the microarray suggests that a general downregulation of gene expression may occur in the retina–RPE as well as choroid after four days of recovery from induced myopia. It should be noted that the chick gene microarray used in the present study represented 4,000 genes with an emphasis on genes expressed in the chicken immune system and therefore does not provide a comprehensive list of all changes in gene expression that might be occurring in the retina, RPE, or choroid during the recovery from induced myopia. Additionally, since microarray analyses were performed on RNA pooled from three complex tissues (retina, RPE, and choroid), the possibility exists for false negatives due to opposite, mutually canceling effects of gene expression in two or more cell types contained in these ocular tissues. Moreover, the use of the contralateral untreated eye as the control eye may result in additional false negatives in our analyses, as some aspects of the ocular response to imposed plus defocus are also observed in the contralateral untreated eyes [32–35].

Due to the uniquely increased gene expression of *ATH* in the retina–RPE–choroid of eyes following one day of recovery as detected by microarray analysis, the present study was aimed at evaluating the protein expression of ATH in chick ocular tissues of normal eyes, form-deprived eyes, recovering eyes, and contralateral eyes. ATH was originally identified as a thymus-specific antigen [32] that is synthesized and secreted by epithelial cells in the thymic cortex [30,36]. Based on its protein and gene sequences [37,38], ATH is described as a parvalbumin having a molecular weight of 11.7 kDa and three helix–loop–helix (EF-hand) motifs, two of which are functional for binding calcium [39,40]. On the basis of their metal ion-binding properties, parvalbumins are generally considered cytosolic Ca^2+^ buffers and transporters, and play a role in skeletal muscle contraction [41] and neuronal de-excitation [42]. ATH has also been shown to function in T-cell maturation and differentiation in the thymus in a paracrine manner [43,44]. ATH has been shown to induce the expression of T cell surface markers in bone marrow precursor cells [45] and enhance the graft-versus-host reaction in chick embryos treated with peripheral blood mononuclear cells previously incubated with ATH, possibly due to activation of γδ T lymphocytes [31].

Steady-state mRNA concentrations of *ATH* relative to that of the housekeepjng gene *GAPDH*, were compared in chick ocular tissues using quantitative real time reverse transcription PCR. *ATH* mRNA levels were lowest in the retina–RPE and approximately tenfold higher in the choroid. *ATH* expression was orders of magnitude higher in the sclera (roughly 390 fold) and extraocular muscle (roughly 900 fold) as compared with that in the retina–RPE.

Following confirmation of *ATH* gene expression in chick ocular tissues, we evaluated ATH protein expression using a specific monoclonal antibody generated against chick ATH [30]. ATH was detected in the retina, choroid, suprachoroidal fluid, sclera, and extraocular muscle, but little to no ATH was detected in serum or brain. Confocal microscopy enabled the detection of abundant levels of ATH throughout the choroidal stroma, choriocapillaris, and in the choroidal side of Bruch’s membrane. Interestingly, the fibrous layer of the chick sclera was intensely positive for ATH, while the cartilaginous layer of the sclera was negative. It has been suggested that the fibrous layer of the chick sclera is comparable to the mammalian sclera, while the cartilaginous sclera is lost through evolution [46–48]. The finding of ATH exclusively in the fibrous sclera allows for speculation that ATH (or the mammalian ortholog) may play a role in the growth and remodeling of the mammalian sclera. Specific immunolabeling for ATH was also detected in the cell bodies and synaptic processes of a subset of neurons largely located on the inner 1/3 to 1/2 of the INL with processes extending into several sublaminae of the IPL. The retinal distribution of ATH is similar to that previously described for parvalbumin in the chick retina [49], where parvalbumin-immunoreactive amacrine cells located in the inner margin of the INL could be seen branching to multiple lamina of the IPL. The distribution of ATH in the chick retina is also similar to that of bistratified rod amacrine cells (AII) amacrine cells recently described in the bat retina [50], although it is unlikely that ATH-positive neurons are AII amacrine cells, as AII amacrine cells have not been previously described in the chicken. The size and location of the ATH-immunoreactive cells are also similar to that described for enkephalin-, neurotensin- and somatostatin-like immunoreactive (ENSLI) amacrine cells in the chicken retina [51,52]. Interestingly, ENSLI cells have been demonstrated to have a light-dependent pattern of activity [53] which has been shown by one laboratory to be suppressed by visual form deprivation [54], but was not confirmed by others [55]. Comparison of ATH levels in ocular tissues and suprachoroidal fluid of treated and control eyes indicated that ATH protein levels were significantly elevated in the choroids of chick eyes following four days of recovery as compared with contralateral control eyes. These results suggest that the increased *ATH* mRNA levels detected by microarray analyses of retina–RPE–choroid of chick eyes following one day of recovery likely represent changes in *ATH* gene expression exclusively in the choroid. Interestingly, ATH levels returned to levels similar to controls by seven days of recovery, suggesting a turnover of ATH from the choroidal stroma. The finding of ATH in the suprachoroidal fluid also suggests that ATH is circulated between the choroidal stroma and the choroidal lymphatic channels in chick eyes during all ocular growth states; however, it is also possible that cellular contaminants obtained during the collection of suprachoroidal fluid may have contributed to the ATH protein levels observed in the suprachoroidal fluid. The peak of ATH accumulation in the choroid following four days of recovery corresponds to the significant increase in choroidal permeability [8,10] and choroidal thickness [7,9] previously observed. It is unlikely that the increase choroidal levels of ATH is a reflection of increased permeability of serum derived ATH, as immunolabeling for ATH in the choroidal stroma was intense even when chicks were perfused in vivo with PBS to clear tissues of serum. Moreover, levels of ATH in chick serum were barely detectible by western blot analysis in this study.

The increased expression of choroidal ATH does correlate with the rapid decrease in scleral proteoglycan synthesis observed during the deceleration of ocular elongation that occurs during the recovery from myopia [9,22]. It is therefore tempting to speculate that secreted ATH from the choroid may regulate scleral extracellular matrix changes that occur during the recovery from myopia. However, preliminary experiments testing the direct effect of ATH on scleral proteoglycan synthesis in vitro suggest that ATH has no effect on scleral proteoglycan synthesis (data not shown).

Oncomodulin, a mammalian protein homologous to ATH (54% identity) [56], has been suggested to be synthesized and secreted by macrophages. Oncomodulin has been suggested to act as a potent growth and regeneration factor for retinal ganglion cells and peripheral sensory neurons [57], although a more recent study indicates that this is not the case [58]. It is therefore possible, that circulating macrophages may accumulate in extravascular locations in the choroid during periods of increased permeability and secrete ATH throughout the choroidal stroma.

In summary, the results of the present study show that *ATH* is expressed in the chick retina, choroid, sclera, and extraocular muscle, with highest mRNA and protein expression levels in the sclera and extraocular muscle. Microarray and western blot results suggest that *ATH* mRNA and protein expression are increased by choroidal cells early during recovery from induced myopia, when ocular growth rates are rapidly decelerating. Although the function of ATH in the chick choroid is unknown, based on its high affinity for Ca^2+^ and its role as an endogenous Ca^2+^ buffer [59] it is possible that ATH may participate in the contraction/relaxation of extravascular smooth muscle located in the choroidal stroma or be involved in modulation of synaptic transmission within the network of intrinsic choroidal neurons. We speculate that regulation of these functions may play a role in modulating choroidal thickness and permeability during the recovery from induced myopia [60,61]. Although ATH protein levels were similar in the retina–RPEs of normal, myopic, recovering, and contralateral eyes, the distinct immunolabeling pattern of relatively few neurons in the inner portion of the INL may provide a useful marker for this population of cells in future immunocytochemical studies of chick retinas in a variety of ocular growth states.

## Appendix 1. Summary of microarray analysis of retina–RPE–choroids during recovery from myopia.

To access the table, click or select the words “Appendix 1.” This will initiate the download of a pdf that contains the table.
